# A rare cause of generalized seizures: agenesis and Lipoma of the corpus Callosum

**DOI:** 10.11604/pamj.2014.19.384.5896

**Published:** 2014-12-17

**Authors:** Ahmed Belkouch, Abdelilah Mouhsine

**Affiliations:** 1 Emergency Department,Military Hospital of Instruction Mohamed V, Faculty of Medicine and Pharmacy, Rabat, Morocco; 2 Department of Radiology, Military Hospital of Avicenna, Faculty of Medicine and Pharmacy, Marrakech, Morocco

**Keywords:** Corpus Callosum, Lipoma, seizures

## Image in medicine

**Figure 1:**
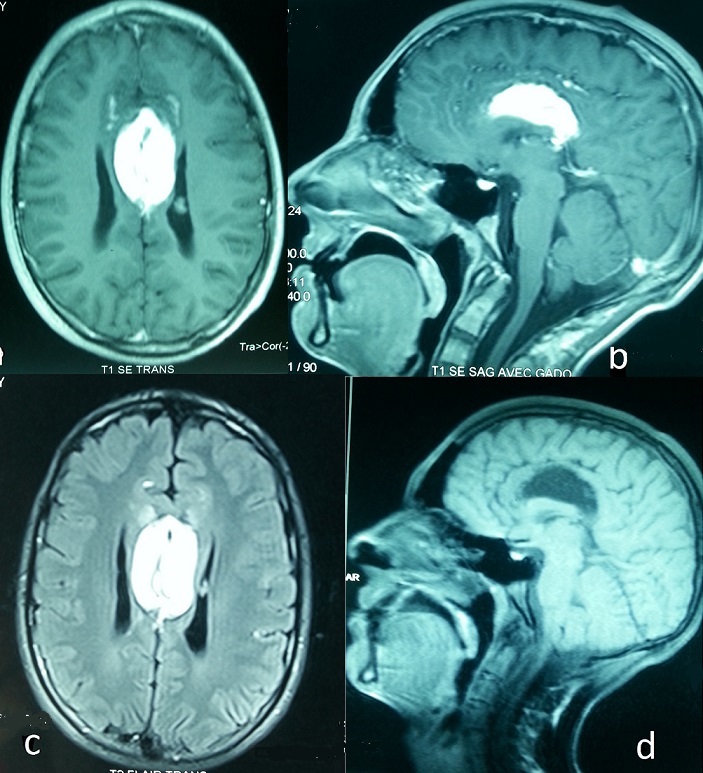
A) MRI on axial cut T1 revealing the interhemispheric high signal lesion; B) MRI on sagittal cut T1 after gadolinium injection showing the same unchanged lesion; C) MRI on axial cut T2 flair revealing the interhemispheric high signal lesion; D) MRI in sagittal cut T1 fat sat showed an hyposignal lesion

An 18 years old young patient with no history presented to the emergency department suffering from episodes of generalized seizures and atypical headache without fever, the onset of symptoms dated back to two months. The clinical examination was normal. CT scan showed an inter hemispheric fat density ovoid formation, measuring about 40x30cm along major axis, compressing the ventricular junctions on both sides with suspected partial agenesis of the corpus callosum (Figure 1a, b, c, d). MRI confirmed the partial agenesis of the corpus callosum (body and splenium); the lipoma was in high fat signal in T1 and T2 and was not modified by injection of gadolinium. This lesion showed arcuate peripheral calcifications, it was also hypointense on SPT1FAT SAT. The patient was stabilized using antiepileptic drugs. Intra cranial lipomas are rare benign tumors, its association with the agenesis of corpus callosum is also rare; the pathogenesis of such malformation is the premature disjunction of the neural ectoderms and skin before the closure of the neural tube. Epilepsy is the most common presentation of the Lipoma of the Corpus Callosum. It can reveal as a generalized seizures as for our patient but also as status epilepticus, partial motor or complex partial seizures. CT scan and especially MRI are the gold standard to diagnose these tumors. Surgical treatment has no place in this case, because of the anatomical relationship with adjacent structures especially with the anterior cerebral arteries which can be damaged.

